# Fungal and Bacterial Communities in the Rhizosphere of *Pinus tabulaeformis* Related to the Restoration of Plantations and Natural Secondary Forests in the Loess Plateau, Northwest China

**DOI:** 10.1155/2013/606480

**Published:** 2013-12-25

**Authors:** Hong-Xia Yu, Chun-Yan Wang, Ming Tang

**Affiliations:** College of Forestry, Northwest A&F University, Yangling, Shaanxi 712100, China

## Abstract

Chinese pine (*Pinus tabulaeformis* Carr.) is widely planted for restoration in destroyed ecosystems of the Loess Plateau in China. Although soil microbial communities are important subsurface components of the terrestrial ecosystems, little is known about fungal and bacterial communities in the rhizosphere of planted and natural *P. tabulaeformis* forests in the region. In this study, fungal and bacterial communities in the rhizosphere of *P. tabulaeformis* were analyzed by nested PCR-DGGE (denaturing gradient gel electrophoresis). Diversity analysis revealed that the values of the Shannon-Wiener index (*H*) and the Simpson index (*D*) of fungal communities were higher in natural secondary forests than in plantations except for the 3-year-old site. Moreover, the values of species richness, *H*, and *D* of the bacterial communities were also higher in the former. Totally, 18 fungal and 19 bacterial DGGE band types were successfully retrieved and sequenced. The dominant fungi in the rhizosphere of *P. tabulaeformis* belonged to the phylum of Basidiomycota, while the dominant bacteria belonged to the phylum of Proteobacteria. Principal component analysis indicated that fungal and bacterial species were more unitary in plantations than in natural secondary forests, and the majority of them were more likely to appear in the latter. Correlation analysis showed no significant correlation between the fungal and bacterial community diversities.

## 1. Introduction

Land degradation and vegetation deterioration caused by human population pressure are growing problems in China. The annual increasing areas of eroded and desertified land are approximately 10 000 and 2 500 km^2^, respectively [1]. One of the most severely affected areas is the Loess Plateau in northwestern China. The present lost area in this region is about 450 000 km^2^ [2], accounting for 72% of the total area (624 000 km^2^) [3]. To accelerate ecological rehabilitation and improve ecological environment in this region, extensive restoration projects have been performed by the Chinese Central Government over the past decades [4]. Forest plantation and natural secondary forest are the two common and important patterns adopted in the ecological restoration and reconstruction. Chinese pine (*Pinus tabulaeformis* Carr.) represents the most predominant pioneer tree species for artificial reforestation and is widely planted due to its high stress tolerance to cold, drought, and poor quality of soil in the Loess Plateau of northwest China [4–6]. On the other hand, the *Pinus* species appear in the early stage of forest succession and form the pioneer forest in natural succession [7, 8].

Soil microorganisms are important subsurface components of terrestrial ecosystems because they play a central role in nutrient cycling as important decomposers [5]. Among them, fungi can also play a key role in restoration processes of soil ecosystem and contribute to soil structures by creating microaggregation of soil particles, thereby improving soil aeration and moisture retention thus enhancing erosion resistance [9]. In addition, fungi, particularly mycorrhizal fungi, influence restoration by acting as mutualistic symbionts [10]. These symbiotic fungi facilitate water and nutrient uptakes of the host plants, improve plant resistance to pathogens, and facilitate primary succession by enhancing the survival and growth ability of forest plants in unfavorable environments and soil conditions [8, 11]. They are very important to pine forests because pines are perceived as obligate ectomycorrhizal (EM) trees and do not develop normally without EM mutualistic symbiosis [12, 13]. Like mycorrhizal fungi, certain rhizospheric bacteria are ubiquitous members in soil microbial communities and have received special attention due to their exceptional ability of exerting beneficial effects on fungi or plants. For example, mycorrhization helper bacteria (MHB) [14] and plant growth promoting rhizobacteria (PGPR) [15] can enhance the rate of mycorrhiza formation [14] and promote the growth of host plants [16], which are essential for the process of ecological restoration and construction during the early forest establishment. Given that fungi and bacteria play important roles in restoration, that land degradation and vegetation deterioration are frequently accompanied with the destruction of microbial communities in soil, and that habitat restoration of microflora is also in progress as the recovery of surface vegetation, there is a clear need to better understand soil microbial communities in different forest restoration patterns.

The traditional understanding of microbial diversity in ecosystems has been limited by the reliance on culture-based approaches. It has become increasingly clear that such approaches only detect a small fraction of edaphon [17] and their limitations are now widely accepted [18]. Recently, substantial advances have been made in microbial ecology because of the development and application of molecular techniques that have overcome the limitations of traditional methods. A number of molecular techniques have been used to investigate the biodiversity of soil microorganisms, including denaturing gradient gel electrophoresis (DGGE) [6], automated rRNA intergenic spacer analysis (ARISA) [19], terminal restriction fragment length polymorphism (T-RFLP) [20], amplified fragment length polymorphism (AFLP) [21], random amplified polymorphic DNA (RAPD) analysis [21], single strand conformation polymorphism (SSCP) [22], temperature gradient gel electrophoresis (TGGE) [23], and oligonucleotide fingerprinting of rRNA genes (OFRG) [24]. After the DNA extraction, the use of polymerase chain reaction (PCR) and community profiling techniques can directly detect the presence of microbial taxa in environmental samples and greatly facilitates the understanding of soil microbial communities.

DGGE was first introduced by Muyzer et al. [28] for microbial community analysis and now is widely used in the analysis of microbial communities in restorations [6, 29–31]. Despite the fact that many studies focus on compositional changes of soil microbial communities in restorations worldwide, there is relatively little information on the change and recovery of fungal and bacterial communities during the restorations on the Loess Plateau in China, where a special ecosystem takes form in the process of ecological restoration and reconstruction [6]. Hence, structure, composition, and taxonomic diversity of fungal and bacterial communities in the region need to be studied urgently. In the present study, DGGE was chosen as the fingerprinting method because it can provide a rapid, visual indication to variations in microbial community structure and individual bands can be excised, cloned, and sequenced in an attempt to identify their origin. The objective of this study was to analyze the communities of fungi and bacteria, as well as their relationship in the rhizosphere of *P. tabulaeformis* within the forest plantations and natural secondary forests on the Loess Plateau of China, and to determine whether forest plantations could develop soil microbial communities as productive and diverse as those found in natural secondary forests.

## 2. Materials and Methods

### 2.1. Field Site and Sampling Procedure

The study site is located in the Lianjiabian Forest of the northern Ziwuling Region, Heshui County, Gansu Province, China (108°10′–109°18′ E, 35°03′–36°37′ N). It is a typical hilly and gully region in the Loess Plateau with an altitude above sea level of 1211–1453 m, and the soil type is calcareous cinnamon soil. This region has a midtemperate continental monsoon climate with an annual average temperature of 7.4°C and annual average precipitation of 587.6 mm. During the Ming and Qing Dynasties, human activities and war destruction made the forests in this region completely devastated and the lands became deserted [5, 32]. In time, vegetation in this abandoned land naturally rehabilitated to the currently existing secondary forests, and the Ziwuling region is one of the best conserved areas in the Loess Plateau with relatively natural secondary forests. On the other hand, from the 1960s [32] ecological restoration and reconstruction were conducted in this region by using *P. tabulaeformis* as the dominant tree species. The covering area of *P. tabulaeformis* forest plantations is approximately 53 000 ha, occupying 81% of the total area of plantation forest [5].

In 2010, a sample plot (20 × 20 m^2^) was established randomly in each of the 3-, 12-, and 25-year-old forest plantations, and another three sample plots were established in respective the 3-, 12-, and 25-year-old natural secondary forests. At each of the six plots, four *P. tabulaeformis* individuals were randomly selected, whereafter, rhizospheric soil (transect depth of 5–20 cm) was sampled according to the methods described by Kidd et al. [33]. All soil collected from the same sampling plot was mixed together equally and as one sample. Soil samples were placed in sealed bags and put into ice box for transport to the laboratory and then stored at −20°C for further analysis.

### 2.2. DNA Extraction and Purification

Total DNA was extracted from 5.0 g rhizospheric soil of each sample according to the procedures described by Zhou et al. [34]. The purification of total soil DNA was carried out by using a TIANgel Midi Purification Kit (Tiangen Biotech Co., Ltd., Beijing, China) as recommended by the manufacturer.

### 2.3. Nested PCR of Fungal and Bacterial Fragments

The ITS region of fungal rDNA gene was amplified using nested PCR. DNA samples extracted from rhizospheric soil were subjected to the first round PCR, using primers ITS1-F [25] and ITS4 [26] (Table 1). The first round PCR products were used as the templates of the second round PCR, using primers ITS1-F with a GC-clamp (40 bases) adhered to its 5′ end and ITS2 [26] (Table 1). All PCR amplifications were carried out in a 50 *μ*L reaction volume, containing 2.5 *μ*L DNA template, 10 mM Tris-HCl (pH 8.3), 50 mM KCl, 2.5 mM MgCl_2_, 0.25 mM dNTP, 0.2 *μ*M of each primer, and 1.25 units Taq DNA polymerase. For both amplification rounds in nested PCR, the same PCR cycling parameters were performed with a S1000 thermal cycler (Bio-Rad, USA) as follows: 94°C for 5 min, then 35 cycles of 95°C for 45 s, 55°C for 45 s, and 72°C for 45 s, and a final extension of 72°C for 10 min.

The variable V3 region of bacterial 16 S rDNA gene was amplified using nested PCR. DNA samples extracted from rhizospheric soil were subjected to the first round PCR, using primers fD1 and rP1 [27] (Table 1). The first round PCR products were used as the templates of a second round PCR, using primers 341f with a GC-clamp (40 bases) adhered to the 5′ end and 534r [28] (Table 1). All PCR amplifications were performed in a volume of 20 *μ*L, containing 1 *μ*L DNA template, 10 mM Tris-HCl (pH 8.3), 50 mM KCl, 2.5 mM MgCl_2_, 0.25 mM dNTP, 0.2 *μ*M of each primer, and 1 unit Taq DNA polymerase. The products were amplified in S1000 thermal cycler (Bio-Rad, USA) under the following conditions: in the first round, 94°C for 5 min, then 30 cycles of 94°C for 1 min, 55°C for 1 min, and 72°C for 1.5 min, and a final extension of 72°C for 5 min; in the second round, 94°C for 5 min, then 30 cycles of 94°C for 30 s, 55°C for 30 s, and 72°C for 30 s, and a final extension of 72°C for 5 min.

PCR products were analyzed by 1% (w/v) agarose gel electrophoresis, stained with ethidium bromide (EB), and visualized under UV light. Obtained PCR products were stored at −20°C for subsequent DGGE analysis.

### 2.4. DGGE Analysis

Twenty microliters of fungal and bacterial nested PCR products was used for DGGE to analyze fungal and bacterial communities, respectively. Gel contained 8% (w/v) polyacrylamide (40% solution, acrylamide/bis-acrylamide = 37.5 : 1, w/w). Vertical denaturing gradient was prepared from 30% (12.6 g urea, 12% (v/v) formamide) to 60% (25.2 g urea, 24% (v/v) formamide) for fungi and from 30% (12.6 g urea, 12% (v/v) formamide) to 70% (29.4 g urea, 28% (v/v) formamide) for bacteria [23, 35]. To integrate the nested PCR products into the gel as soon as possible, DGGE was primarily run at 200 V for 8 min and then performed at 70 V for 13 h in 1× TAE buffer at a constant temperature of 58°C. DGGE was done by using the DCode universal mutation detection system (Bio-Rad, Hercules, CA, USA). After being stained with EB, gel was visualized under UV light and then photographed by Gel Doc imaging system (Bio-Rad, Hercules, CA, USA).

### 2.5. Cloning, Sequencing, and Phylogenetic Analysis

All of the detected bands were excised from the DGGE gel under UV light and then were mashed and incubated in 30 *μ*L sterile deionized water at 4°C overnight. After that, PCR products were purified using TIANgel Midi Purification Kit (Tiangen Biotech Co., Ltd., Beijing, China). Purified PCR products from each isolated target band were ligated to the pGEM-T Easy vector (Promega, Madison, WI, USA) and transformed into *Escherichia coli* DH5*α* competent cells by following the manufacturer's protocol. Positive clones were screened from the transformed cells by using blue-white spot procedures according to the method of Gao et al. [36]. Cloned inserts were checked by PCR amplification (PCR reaction system and conditions as described above) using primers ITS1-F and ITS2 for fungi, 341f and 534r for bacteria. The positive clones were sent to Tianyi Huiyuan Bioscience and Technology Inc. (Beijing, China) for sequencing.

To confirm the origin of rhizospheric microbial rDNA gene sequences, obtained sequences were analyzed by BLAST (basic local alignment search tool) and compared with sequences deposited in the GenBank database at the National Center for Biotechnology Information (Bethesda, USA). The best representative of each individual DGGE band type was deposited in GenBank database under accession numbers KF673104 to KF673140. Phylogenetic relationships of the rhizospheric microbiota were analyzed by constructing phylogenetic trees which contained the sequences obtained by us and database reference sequences. All of these sequences were edited and trimmed manually using BioEdit software (version 7.0.9.0) and aligned by Clustal X 1.81. Finally, the neighbor-joining trees were constructed by using MEGA version 5.05 with the Kimura two-parameter model [37]. To determine the support for each clade and assess the reliability of the branching pattern, bootstrap analysis was performed using 1000 replications.

### 2.6. Statistical Analysis

DGGE images were digitalized and analyzed using Quantity One software 4.6.2 (Bio-Rad, Hercules, CA, USA). Presence or absence of the bands in each lane of the DGGE gel was converted to a binary matrix. After that, the data were subjected firstly to detrended correspondence analysis (DCA) to decide on the response model (linear or unimodal) of ordination. The result showed that the max length gradients were 1.704 and 1.694 for fungi and bacteria species, respectively. Therefore, principal component analysis (PCA) was chosen for inferring correlations between sample plots and communities of rhizospheric microbiota. PCA was performed using the Canoco version 4.5 (Centre for Biometry, Wageningen, The Netherlands), and a Monte Carlo permutation test with 499 replicates was permuted using cyclic shifts. Based on the number and intensity of bands in DGGE profiles, species richness (*S*), Shannon-Wiener index (*H*), Evenness index (*E*_*h*_), and Simpson index (*D*) were calculated in accordance with the following formula [38, 39]:

(1) $$\begin{array}{c} H=-\overset{S}{\underset{i=1}{\sum }}\left(\frac{Ni}{N}\right)\ln\left(\frac{Ni}{N}\right),\\ E_{h}=\frac{H}{\ln S},\\ D=\overset{S}{\underset{i=1}{\sum }}{\left(\frac{Ni}{N}\right)}^{2}, \end{array}$$

where *Ni* was the peak density of the *i*th band, *N* was the sum of the peak density of all bands in a lane, and *S* was the total band number in a lane. The diversity indices (*S*, *H*, *E*_*h*_, and *D*) of fungi and bacteria were used for the correlation analysis with SAS version 8.1 (SAS Institute Inc., Cary, NC).

## 3. Results

### 3.1. Amplification of Fungal and Bacterial DNA by Nested PCR

After the first round PCR amplification, it was difficult to detect fungal and bacterial DNA bands in the agarose gel. However, the target fungal and bacterial fragments could be detected clearly after the second round PCR amplification (shown in Supplementary Material available online at http://dx.doi.org/10.115/2013/606480), demonstrating that target fragments could be amplified by nested PCR.

### 3.2. DGGE Profiles of Fungal and Bacterial Communities

**Figure 1:**
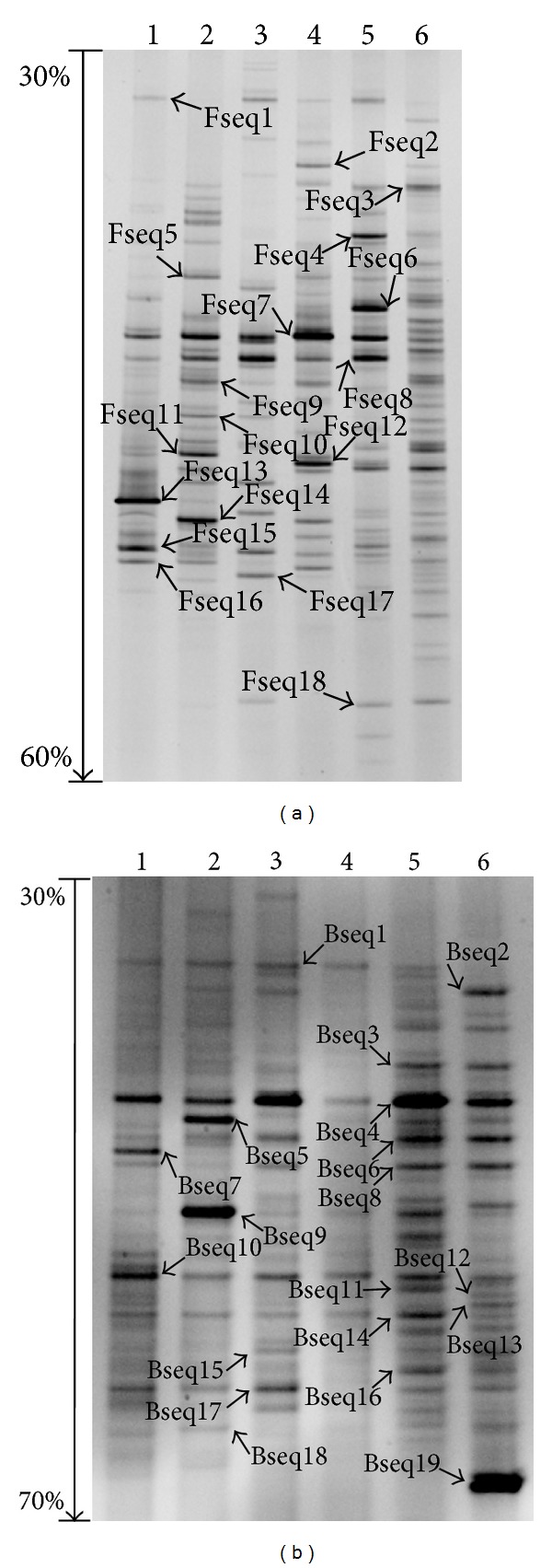
DGGE patterns of fungal and bacterial communities in the rhizosphere of* Pinus tabulaeformis*. (a) Fungal community structure after PCR amplification using primer pair ITS1-F-GC/ITS2 and DGGE. (b) Bacterial community structure after PCR amplification using primer pair 341f-GC/534r and DGGE. Lanes 1–3 represent the samples collected from 3-, 12-, and 25-year-old *P. tabulaeformis* forest plantation, and lanes 4–6 represented the samples collected from 3-, 12-, and 25-year-old *P. tabulaeformis* natural secondary forest, respectively.

The compositions of fungal and bacterial communities in the rhizosphere of *P. tabulaeformis* were compared by nested PCR-DGGE. Differences among lanes in both DGGE profiles of fungal and bacterial communities were clearly observed and there were some striking similarities between the two profiles (Figure 1). Firstly, the structures and compositions of fungal and bacterial communities varied between the two restoration patterns. Whereas several bands in DGGE profiles were common between the two restoration patterns, some bands were unique. Secondly, the structures and compositions of fungal and bacterial communities in the same restoration pattern varied within different sample sites. Thirdly, the two restoration patterns shared most of the species. Furthermore, the signal intensity of DNA band types was variable, from strong to weak.

### 3.3. Diversity of Fungal and Bacterial Communities

The diversity indices (*S*, *H*, *E*_*h*_, and *D*) of fungal and bacterial communities were calculated (Table 2). The diversity of fungi was different between the two restoration patterns. The values of *H* and *D* were higher in natural secondary forests than those in forest plantations except for the 3-year site. The values of*S*, *H*, and *D* reached the highest in the 12-year site of natural secondary forest. The value of *E*_*h*_ changed very little among all study sites and was higher in natural secondary forests than in forest plantations.

The diversity of bacteria was also different between the two restoration patterns. The values of *S*, *H*, and *D* were higher in natural secondary forests than in forest plantations and reached the highest in the 25-year site of natural secondary forest. The value of *E*_*h*_ was lower in natural secondary forests than in forest plantations except for the 25-year site.

The values of *S* and *H* were higher for bacteria than fungi with the exception of the 3-year site. By contrast, the value of *E*_*h*_ was lower in bacterial communities than in fungal communities. In addition, for both bacteria and fungi, the value of *D* showed no obviously regular change between the two restoration patterns.

### 3.4. Phylogenetic Analysis and Microbial Taxon Identification

**Figure 2:**
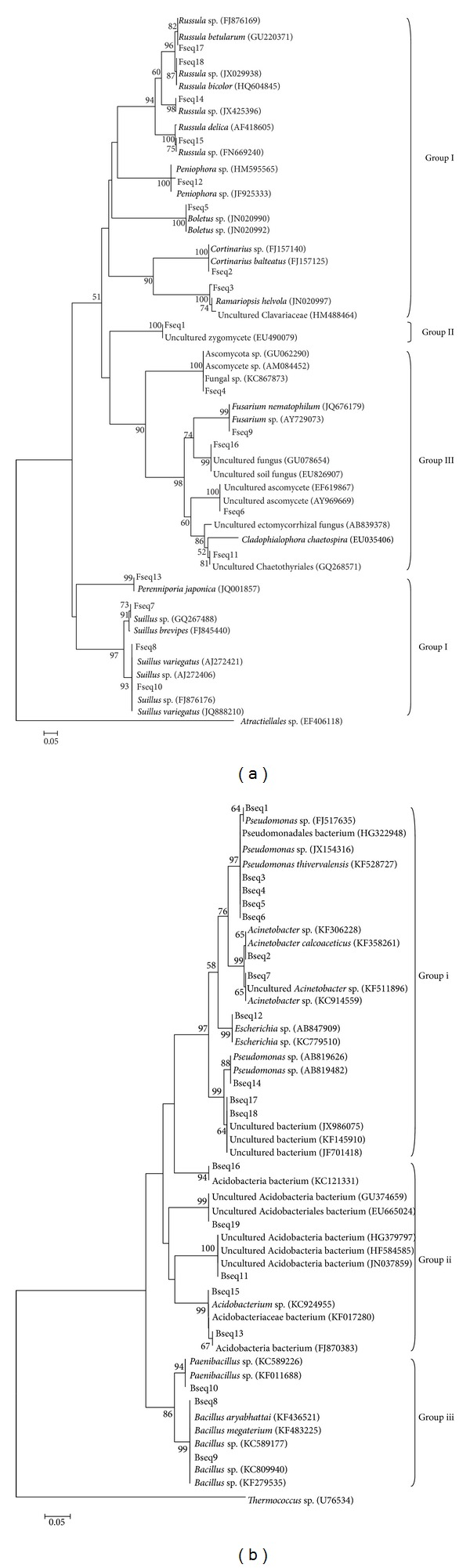
Neighbor-joining phylogenetic tree of fungi and bacteria in the rhizosphere of *Pinus tabulaeformis* based on their partial ITS and 16 S rRNA sequences, respectively. (a) Fungal community composition by recovering rDNA gene sequences from the DGGE gel and conducting phylogenetic analyses. (b) Bacterial community composition by recovering 16 S rDNA gene sequences from the DGGE gel and conducting phylogenetic analyses. Only bootstrap analysis was performed using 1,000 replicates. Bootstrap values above 50% were shown.

By recovering rDNA gene sequences from DGGE gels and conducting phylogenetic trees, we were able to identify some of the fungi and bacteria presented at the study sites (Figure 2). All of the obtained fungal sequences Fseq ranged from 217 to 321 bp and belonged to three groups. Twelve of the eighteen species were clustered into the clade of group I, Basidiomycota, including Fseq2 that belonged to the genus *Cortinarius*, Fseq3 that belonged to the genus *Ramariopsis*, Fseq5 that belonged to the genus *Boletus*, Fseq7, Fseq8, and Fseq10 that belonged to the genus *Suillus*, Fseq12 that belonged to the genus *Peniophora*, Fseq13 that belonged to the genus *Perenniporia*, and Fseq14, Fseq15, Fseq17, and Fseq18 that belonged to the genus *Russula*. The Fseq1 was classified as group II, Zygomycota. Moreover, Fseq4, Fseq6, Fseq9, Fseq11, and Fseq16 were clustered into the clade of group III, Ascomycota.

The results of sequenced bacteria indicated that all the obtained sequences Bseq belonged to three groups, and sequence size ranged from 169 to 195 bp. Most of bacteria species (eleven of the nineteen identified species in total) belonged to *Pseudomonas*, *Acinetobacter*, and *Escherichia* in group (i), and group (i) was Proteobacteria. Eight of the eleven species were clustered into the clade of *Pseudomonas*. The Bseq11, Bseq13, Bseq15, Bseq16, and Bseq19 were classified as group (ii), Acidobacteria. Among them, Bseq15 was identified as *Acidobacterium* sp. with the high similarity (100%). Group (iii) was Firmicute, including Bseq8 and Bseq9 identified as *Bacillus* sp. with high confidence, and Bseq10 was closely related to *Paenibacillus* sp. in Bacillales.

### 3.5. PCA and Correlation Analysis

**Figure 3:**
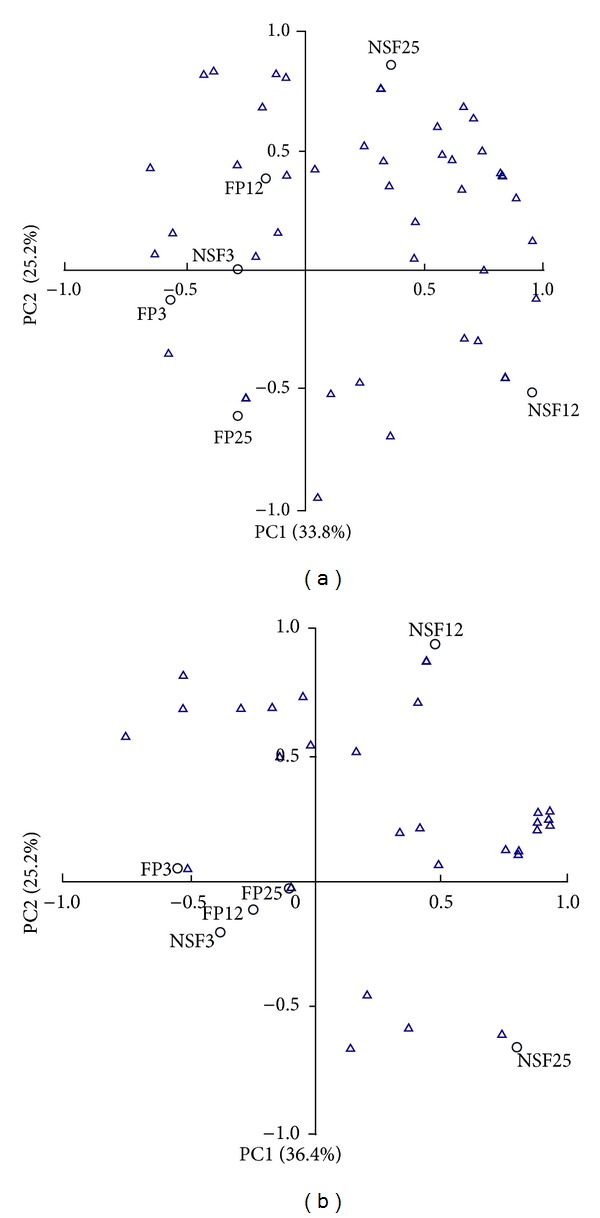
PCA depicted relationship between fungal (a) and bacterial (b) species in the rhizosphere of *Pinus tabulaeformis* and sampling sites. Open triangles indicated microbial species.

PCA analysis was used to assess relationships between sample plots and communities of rhizospheric microbiota (Figure 3). When the fungal and bacterial communities were analyzed by PCA, the first two axes explained 61.6% and 59.0% of the variation in the data, respectively. The microbial communities in the rhizosphere of *P. tabulaeformis* showed some of the similarities between fungi and bacteria, in accordance with the result of DGGE profiles. The six sample sites were divided into four quadrants by axes of principal component 1 (PC1) and principal component 2 (PC2). The distribution of sites was divided into two groups by PCA axis; sites FP3, FP12, FP25, and NSF3 were in group one on the left part of PCA, while sites NSF12 and NSF25 were in group two on the right part of PCA. The majority of fungal and bacterial species were also represented on the right part of the PCA ordination diagram, indicating that they were more likely to appear in sites NSF12 and NSF25. Moreover, the distribution of sites was more compact in forest plantations than in natural secondary forests, indicating that microbial species were more unitary in forest plantations than in natural secondary forests.

In order to understand the relationship between fungal and bacterial communities, correlation analysis was applied. No significant correlation was observed between the diversity of fungal and bacterial communities, as well as between the two restoration patterns. However, there were correlations within the community diversity indexes of fungi, as well as bacteria. For fungi, *S* was significantly and positively correlated with *H* (*r* = 0.995, *P* < 0.01) and *D* (*r* = 0.980, *P* < 0.01), and *H* also had significantly positive correlation with *D* (*r* = 0.995, *P* < 0.01). As to bacteria, *S* showed significantly positive correlation with *H* (*r* = 0.996, *P* < 0.01) and *D* (*r* = 0.978, *P* < 0.01) and exhibited a positive correlation with *E*_*h*_ (*r* = 0.851, *P* < 0.05). Moreover, *H* was also positively correlated with *E*_*h*_ (*r* = 0.832, *P* < 0.05) and *D* (*r* = 0.993, *P* < 0.01).

## 4. Discussion

### 4.1. Phylogenetic Analysis and Dominant Taxa

The fungal communities in the rhizosphere soil samples of *P. tabulaeformis* collected from plantations and natural secondary forests were distributed across three groups, Basidiomycota (66.67%), Ascomycota (27.78%), and Zygomycota (5.56%). This was consistent with previous investigations in pine forest soils [40, 41]. It was easy to see that Basidiomycota was the most important fungal phylum in the *P. tabulaeformis* rhizospheric soil of these forests. Nie et al. [42] also compared fungal communities between natural and planted pine forests and revealed that Basidiomycota was the dominant phylum of fungi. However, He et al. [40] detected mostly Zygomycota species from soil of adjacent natural forests and hoop pine (*Araucaria cunninghamii*) plantation ecosystems. In the present study, most fungi belonging to the phylum Basidiomycota were fitted into already described species. By contrast, most fungi belonging to Ascomycota and Zygomycota were classified as uncultured or unidentified fungi. These unidentified fungal sequences might indicate that some unknown fungal groups exist in these soils. Hence, more intensive sampling and high-throughput sequencing experiments are needed to describe fungal communities in the two forest restoration soils.

Matsuda and Hijii [43] revealed that russuloid species were the most frequent and dominant EM fungi in forests. In this study,* Russula* of the order of Russulales was the dominant genus in the rhizospheric soil of *P. tabulaeformis* collected from plantation and natural secondary forests, followed by *Suillus* of the order of Boletales. Species that belonged to these two genera were known as EM fungi and could form EM symbiotic associations with plants, especially pines [42, 44]. EM fungi could provide buffering capacity and promote the growth and survival of forest plants to resist unfavorable environmental and soil conditions [45]. Mycorrhizal fungi are known as essential components of a self-sustaining ecosystem [45], and their presence implies that the recovery of fungi along with the process of forest restorations and ecosystem in this region were improving.

The bacterial communities in the rhizospheric soil samples of *P. tabulaeformis* collected from plantations and natural secondary forests were also distributed across three groups, Proteobacteria (57.89%), Acidobacteria (26.32%), and Firmicutes (15.79%). It was easy to see that Proteobacteria was the most important bacterial phylum in the *P. tabulaeformis* rhizospheric soil of these forests. This result was in accordance with other recent researches on rhizospheric bacterial diversity. Lottmann et al. [44] reported that Proteobacteria was the dominant phylum of bacteria in the rhizosphere of *P. radiate*. The main bacterial group detected in a study conducted on natural and planted pine forests in China by Nie et al. [42] was also Proteobacteria. Because of the high functional and species diversity and the persistence in oligotrophic environments, Proteobacteria were assumed to occupy and dominate many different niches in unfavourable forest ecosystems [46]. *Pseudomonas* of the phylum Proteobacteria was found as common resident in rhizospheric (particularly mycorrhizospheric) soil of forest ecosystems [47, 48] and was included in groups of soil microorganisms in association with plants and fungi, such as PGPR, MHB, and EMAB (ectomycorrhiza associated bacteria) [49]. The results of the present study indicated that *Pseudomonas* was the most frequently detected genus in the rhizospheric soil of *P. tabulaeformis*. Rózycki et al. [50] have analyzed bacterial communities in soil and the root zone of *P. sylvestris* and also found *Pseudomonas *spp. as being the dominating bacteria. However, Chow et al. [51] characterized the bacterial diversity in rhizospheric soil of *P. contorta* from British Columbia Forest and revealed that the largest cluster of bacteria belonged to *Burkholderia*.

### 4.2. Comparison of Microbial Community Structures between Plantation Forest Restoration Sites and Natural Secondary Succession Fields

According to PCA analyses, microbial species were more unitary in forest plantations than in natural secondary forests, and the majority of fungal and bacterial species were more likely to appear in natural secondary forests. It was generally assumed that forest plantations were negative from the viewpoint of biodiversity conservation, or at least their biodiversities were lower than those of natural forests [52]. One of the main reasons appeared to be the uniformization in plant communities in these forest ecosystems. Several studies indicated that plant community may affect microbial biodiversity and community composition [10, 53].

Although previous studies have shown that the diversity of microbial communities can be affected by restoration patterns [42, 46], the correlation analysis showed no correlation between restoration patterns and the diversity of fungal and bacterial communities in this study. This indicates that microbial communities may actually be more affected by the plant species [10, 53] and soil factors [52] rather than by restoration patterns. In addition, despite the fact that microbial communities were changed with the process of restoration in planted or natural forests, correlation analysis in the present study showed no correlation between the restoration age and the community diversities of fungi and bacteria. Nonetheless, previous studies indicated different results and revealed that forest age was associated with microbial diversity [54, 55]. Although the exact reasons for this contradicting result remain elusive, one possible explanation may be that the temporal scale in the present study was too short, so there was no consistency in age of forest restoration and microbial habitats.

The result of DGGE profiling and PCA analysis showed some similarities between fungal and bacterial communities in the rhizosphere of *P. tabulaeformis*. However, no significant correlation was observed between the diversity indices of fungal and bacterial communities. The correlation between them may be dependent on the type of fungi or bacteria studied. Cavagnaro et al. [56] indicated that mycorrhizal fungi had no effect on the community structures of ammonia oxidizing bacteria. But Zhang et al. [6] showed that mycorrhizal communities had a significant positive correlation with bacterial communities in the Loess Plateau. In addition, there were significantly positive correlations among the diversity indices of fungal communities, as well as for the diversity indices of bacterial communities. Zhang et al. [6] revealed that interactions among fungal and bacterial species may be less frequent than interactions within fungal or bacterial species in the process of planted and natural forest restorations [57]. Interactions within fungal or bacterial species had relatively greater impact on the recovery of microbial communities, which might be due to several factors. First, the same type of microorganisms had the similarities of nutritional needs [58, 59], so there was more nutrient competition than would be in the care for two different types of microorganisms, requiring different nutrients in the process of restorations. Besides, when some mutual symbiotic fungi (e.g., mycorrhizal fungi) or some helpful bacteria (e.g., PGPR) were in symbiosis with plants, they competed not only for nutrition but also for colonization sites and living spaces [13, 60].

## 5. Conclusions

*P. tabulaeformis* is suitable for soil and vegetation restoration of destroyed ecosystems due to its high stress tolerance and conservation of soil and water. According to Liang et al. [7] and Oria-de-Rueda et al. [8], the pine-dominated forests were undeniably a transitional step to a climax state dominated by broadleaved forests in succession. During succession, these coniferous stands protected soil erosion and kept an appreciable microbial diversity, which were important for ecological restoration and sustainable development of the region. Although it was to be expected that coniferous forest plantations would show low community diversities, results of the present study indicated that the diversity indices (*S*, *H*, *E*_*h*_, and *D*) of fungi and bacteria were not absolutely higher in the natural secondary forest sites. The fungal *H* and *D* were higher in natural secondary forests than in forest plantations except for the 3-year site, and the bacterial *S*, *H*, and *D* were higher in natural secondary forests than in forest plantations. However, the fungal *E*_*h*_ contained similar value in the two types of forests and the bacterial *E*_*h*_ was lower in natural secondary forests than in forest plantations except for the 25-year site. Therefore, forest plantations could provide relative rates of microbial community diversities similar to those found in natural secondary forests as well as play an essential role in these unfavorable ecosystems to prevent land degradation and vegetation deterioration on the Loess Plateau in China.

Structure, composition, and diversity of fungal and bacterial communities need to be studied urgently because this is essential to ecological and microbial habitat restoration in land degraded and vegetation deteriorated Loess Plateau in China. To our knowledge, this study is the first study to investigate both fungal and bacterial community structures in the rhizosphere of *P. tabulaeformis*. We compared these microbial communities in planted and natural pine forests on the Loess Plateau of China by applying nested PCR-DGGE method. Our results revealed that some fungal and bacterial species were shared in both forest plantations and natural secondary forests of *P. tabulaeformis*. These species deserve further study due to their potential utility in restoration of destroyed ecosystems on the Loess Plateau of China.

## Figures and Tables

**Table 1:** Primers used for nested PCR amplification of soil fungal and bacterial communities in the rhizosphere of *Pinus tabulaeformis*.

Primer name	Primer sequence (5′-3′)	Reference
Fungi		
ITS1-F	CTTGGTCATTTAGAGGAAGTAA	Gardes and Bruns [25]
ITS4	TCCTCCGCTTATTGATATGC	White et al. [26]
Clamp	CGCCCGCCGCGCGCGGCGGGCGGGGCGGGGGCACGGGGGG	Gardes and Bruns [25]
ITS1-F-GC	Clamp-CTTGGTCATTTAGAGGAAGTAA	Gardes and Bruns [25]
ITS2	GCTGCGTTCTTCATCGATGC	White et al. [26]
Bacteria		
fD1	AGAGTTTGATCCTGGCTCAG	Weisburg et al. [27]
rP1	ACGGTTACCTTGTTACGACTT	Weisburg et al. [27]
341f	CCTACGGGAGGCAGCAG	Muyzer et al. [28]
341f-GC	Clamp-CCTACGGGAGGCAGCAG	Muyzer et al. [28]
534r	ATTACCGCGGCTGCTGG	Muyzer et al. [28]

**Table 2:** Richness (*S*), Shannon-Wiener index (*H*), Evenness index (*E*_*h*_), and Simpson index (*D*) of fungal and bacterial communities in the rhizosphere of *Pinus tabulaeformis*. FP represents forest plantation, and NSF represents natural secondary forest.

Forest restoration pattern	Site (years)	Species richness (*S*)	Shannon-Wiener index (*H*)	Evenness index (*E*_*h*_)	Simpson's index (*D*)
Fungi					
FP	3	15	3.892	0.996	0.932
	12	18	4.143	0.994	0.942
	25	19	4.235	0.997	0.946
NSF	3	13	3.696	0.999	0.923
	12	25	4.635	0.998	0.960
	25	19	4.242	0.999	0.947
Bacteria					
FP	3	15	3.790	0.970	0.922
	12	27	4.656	0.979	0.957
	25	21	4.237	0.965	0.941
NSF	3	20	4.183	0.968	0.939
	12	30	4.778	0.974	0.960
	25	38	5.219	0.988	0.972
